# Pulmonary sclerosing hemangioma presenting with dense spindle stroma cells: a potential diagnostic pitfall

**DOI:** 10.1186/1746-1596-7-174

**Published:** 2012-12-10

**Authors:** Xu-Yong Lin, Yan Wang, Chui-Feng Fan, Yang Liu, Juan-Han Yu, Shun-Dong Dai, Liang Wang, En-Hua Wang

**Affiliations:** 1 Department of Pathology, the First Affiliated Hospital and College of Basic Medical Sciences, China Medical University, Shenyang 110001, China; 2 Institute of pathology and pathophysiology, China Medical University, Shenyang 110001, China

**Keywords:** Pulmonary sclerosing hemangioma, Mesenchymal tumor

## Abstract

Pulmonary sclerosing hemangioma (PSH) is an uncommon pulmonary tumor. Histologically, PSH typically consists of two types of cells, surface cuboidal cells and polygonal cells, four architectural patterns including papillary, sclerotic, solid, and hemorrhagic. Herein, we present a case of PSH in a 59-year-old Chinese female. The tumor was predominantly composed of solid area presenting with diffuse spindle cells rather than polygonal cells. Focally, classical papillary and sclerotic area could be seen. Immunohistochemical staining showed that the spindle cells were positive for TTF-1, EMA, Actin(SM) and Vimentin, and negative for cytokeratin, cytokeratin7, cytokeratin5/6, surfactant apoprotein A, surfactant apoprotein B, CD34, CD99, S-100, HMB45, Desmin, Synaptophysin, CD56, ALK and Calretinin. The immunophenotype of the dense spindle cells in this case was similar to that of the polygonal cells, and thus the spindle cells may be the variants of polygonal cells. Based on morphologic features and the immunohistochemical profile, the tumor was diagnosed as a PSH. The significance of spindle cells change is unclear for us. To our knowledge, this is the first reported case of PSH showing dense spindle cells in solid area. This case represents a potential diagnostic pitfall, as it may be misdiagnosed as a mesenchymal tumor such as inflammatory myofibroblastic tumor, synovial sarcoma, solitary fibrous tumor, leiomyoma, or even mesothelioma, especially if the specimen is limited or from fine- needle aspiration.

**Virtual slides:**

The virtual slide(s) for this article can be found here: http://www.diagnosticpathology.diagnomx.eu/vs/1235401622806126

## Background

Pulmonary sclerosing hemangioma (PSH) is a rare pulmonary tumor, first described by Liebow and Hubbel [1]. Its histogenesis has been debated for decades [2,3]. The increasing immunohistochemical and molecular findings indicate that it originates from primitive respiratory ephithelium [4,5]. Although the nature of PSH is still controversial, its histological feature has been well described. Histologically, PSH contains surface cuboidal cells and polygonal cells, and these two types of cells typically form four architectural patterns including papillary, sclerotic, solid, and hemorrhagic [6,7]. The surface cuboidal cells resemble normal type II pneumocytes while the polygonal cells are diffusely distributed in stroma, and have no counterparts in normal lung tissue. The majority of polygonal cells display well-defined borders, abundant clear to eosinophilic cytoplasm with fine chromatin and inconspicuous nucleoli. The mitosis is rarely observed in PSH, while the tumor cells occasionally display moderate to marked cellular atypia. The presence of the two distinct cell types and the mixture of histological patterns may favor the correct diagnosis. Herein, we present a peculiar case of PSH predominantly composed of sheets of spindle cells in a 59-year-old Chinese female. Focally, the typical sclerotic and papillary region comprising cuboidal cells and polygonal cells could be seen. The presence of dense spindle cells may pose a great diagnostic challenge. It may be misdiagnosed as a mesenchymal tumor, especially if the specimen is limited or from fine needle aspiration.

### Case presentation

A 59-year-old female without a history of smoking referred to our hospital for complaining of a chest pain and coughing in the past two days. Computed tomography scan showed that there was a peripheral round, solitary, well-circumscribed nodule in the lower lobule of right lung. The diameter of the nodule was 4.7×4.1 cm. The patient reported that a nodule measuring 2.7×2.0 cm was found two years ago during routine examination. She did not accept any therapy for the past two years since there was no obvious symptom. In the current visit, the patient underwent lobectomy and lymph node dissection in our hospital since intraoperative frozen biopsy failed to absolutely exclude the possibility of a malignancy.

## Materials and methods

The resected specimens were fixed with 10% neutral-buffered formalin and embedded in paraffin blocks. Tissue blocks were cut into 4-μm slides, deparaffinized in xylene, rehydrated with graded alcohols, and immunostained with the following antibodies: cytokeratin (CK), cytokeratin 5/6 (CK 5/6), cytokeratin7 (CK7), surfactant apoprotein A (SPA), surfactant apoprotein B (SPB), Vimentin, CD34, CD99, S100, Desmin, Actin(SM), thyroid transcription factor 1 (TTF-1), EMA, HMB45, Synaptophysin (SY), CD56, Calretinin, Anaplastic lymphoma kinase (ALK), and Ki67. Sections were stained with a streptavidin-peroxidase system (KIT-9720, Ultrasensitive TM S-P, MaiXin, China). The chromogen used was diaminobenzidine tetrahydrochloride substrate (DAB kit, MaiXin, China), slightly counterstained with hematoxylin, dehydrated and mounted. For the negative controls, the primary antibody was replaced with PBS.

## Results

**Figure 1:**
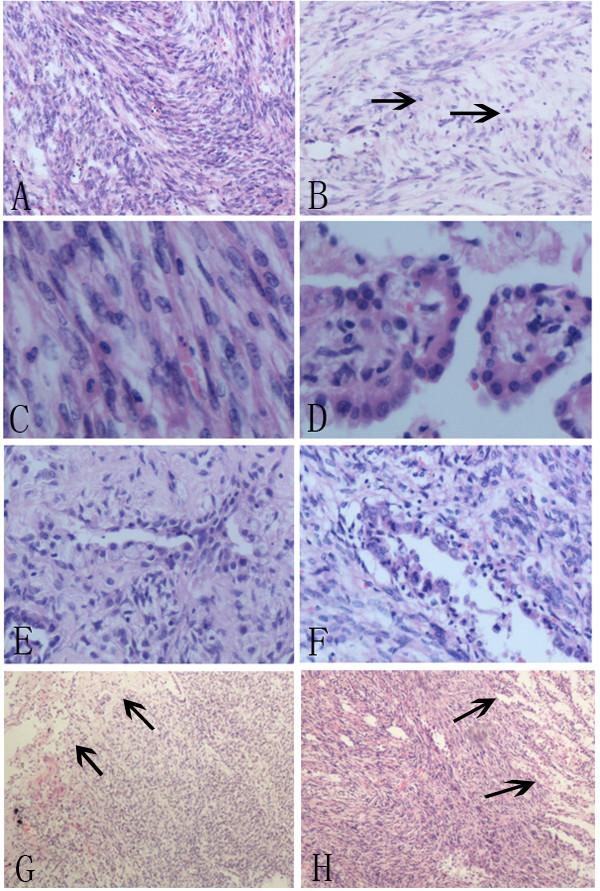
**A, The spindle tumor cells were arranged in bundles and whirling patterns.B**, The scattered lymphocytes (arrows) could be seen in the background of spindle cells. **C**, The spindle cells had no marked cellular atypia, with pale to eosinophilic cytoplasm, fine chromatin and inconspicuous nucleoli. **D**, Papillary area showed the surface cuboidal cells and polygonal cells in the stroma. **E**, Sclerotic area was characterized by hyalinized collagen with scarcely distributed polygonal cells and adenoidal structures consisting of cuboidal cells. **F**, An adenoidal structure covered with cuboidal cells could be seen in spindle cells area. **G**, **H**, The border of the tumor (arrows) was relatively well circumscribed.

Grossly, the tumor measuring 4.5×4.1×3.2cm was well-circumscribed, and the cut surface showed grey-white in color. Histologically, the tumor was predominantly made up of spindle cells which were arranged in solid sheets, bundles or whirling pattern. The spindle cells were bland, with clear cytoplasm, fine chromatin and inconspicuous nucleoli, and no marked cellular atypia could be observed. The mitosis is rare. In focal area, the classical sclerotic and papillary pattern could be seen. The cuboidal cells were arranged in a tight monolayer on the surface of the papillary pattern, while the polygonal cells were distributed in the stroma. The sclerotic pattern showed abundant hyalinized collagen with almost no polygonal cells. A few adenoidal structures consisting of cuboidal cells could also be seen in spindle cells solid area and sclerotic area. In addition, inflammatory cells including lymphocytes, plasmocytes and histiocytes were scattered in all areas (Figure 1). No lymph node metastases were found in excised lymph nodes.

**Figure 2:**
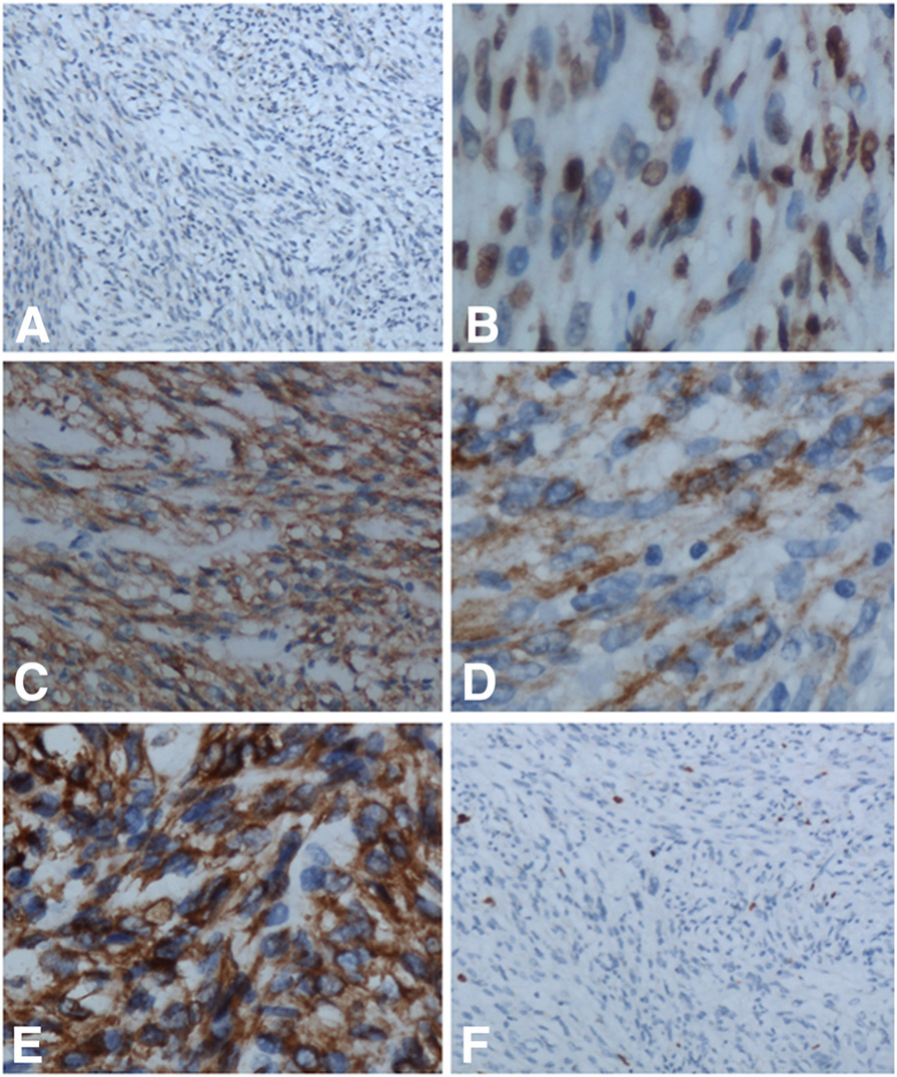
**A, The spindle cells were entirely negative for CK.B**, The majority of the spindle cells were positive for TTF-1. **C**, Diffuse and strong expression of EMA could be seen in the spindle tumor cells. **D**, Focally, expression of Actin(SM) could be seen in the spindle cells. **E**, The spindle cells also showed strong and diffuse expression of Vimentin. **F**, Less than 2% of tumor cells were positive for Ki67.

Immunohistochemical staining showed that the cuboidal cells were positive for TTF-1, EMA, CK, CK7, SPA and SPB; the polygonal cells were positive for TTF-1, EMA and Vimentin. The spindle cells were diffusely positive for TTF-1, EMA and Vimentin, focally positive for Actin(SM) and negative for SPA, SPB, CK, CK 7, CK 5/6, CD34, CD99, S-100, HMB45, desmin, SY, CD56 and Calretinin. Ki67 was expressed in less than 2% of all tumor cells (Figure 2). According to the morphological and immunohistochemical findings, the tumor was diagnosed as a PSH.

## Discussion

PSH is an uncommon lung tumor initially thought to be of vascular origin [1]. However, immunohistochemical and molecular findings strongly suggest that PSH originates from primitive respiratory epithelium [4,5,8]. As an epithelial tumor, PSH is histologically distinctive. It is characterized by two cell types, namely cuboidal cells and polygonal cells, which significantly differ in histological patterns, cellular morphologies, and immunophenotypes. However, in our previous study [4], we performed clonality analysis using the X-linked androgen receptor and phosphoglycerate kinase gene polymorphisms, and the results revealed that the cuboidal cells and polygonal cells had the same cellular origin. The two cell types form a distinct constellation of four architectural patterns including papillary, sclerotic, solid, and hemorrhagic pattern. The correct diagnosis is easily made if one is familiar with the two cell populations and four architectural patterns. Herein, we present a peculiar case of PSH which was predominantly composed of spindle cells. The spindle cells were arranged into solid sheets. Focally, the cells displayed bundles or whirling pattern. The presence of diffuse spindle cells in this case may cause a great diagnostic confusion.

It was well documented that both the cuboidal cells and polygonal cells showed reactivity for TTF-1 and EMA in nearly all cases of PSH. In contrast to the strong expression of CK, CK7, SPA and SPB in cuboidal cells, the polygonal cells usually lack strong expression of the above markers, but show diffuse and strong positivity for Vimentin, and rarely for Actin(SM) [9]. In our case, the cuboidal cells and polygonal cells show consistent staining results with the reports in the literatures [9]. Similar to polygonal cells, the spindle cells were diffusely positive for TTF-1, EMA, and Vimentin, focally positive for Actin(SM); this indicates that the spindle cells may be simply the variants of polygonal cells. However, it is unclear why and when the polygonal cells display the spindle cells change. Moreover, the significance of spindle cells change is also unclear to us.

Although many scholars believe that the tumor is benign, there are reports of lymph node metastasis [10,11], pleural dissemination [12], and metastatic spread to the stomach [13]. So, some authors advocate it may belong to be a tumor with low malignant potential [14]. Dacic et al. [15] proposed that PSH has a similar molecular pathogenesis as bronchioloalveolar carcinoma, so might have a biological behavior similar to BAC. In our case, the size of the tumor increased slowly, no lymph node metastasis was found. But, it is essential for long-term follow-up to further elucidate the clinical behavior of this tumor and significance of spindle cells change.

Because of the extensive presence of spindle cells, a series of mesenchymal tumors including inflammatory myofibroblastic tumor, synovial sarcoma, solitary fibrous tumor, leiomyoma and mesothelioma should be excluded in this case. In addition, the differential diagnosis should also include some uncommon tumors, such as pulmonary epithelioid hemangioendothelioma [16]. Inflammatory myofibroblastic tumor is characterized by the admixture of spindle-shaped and ovoid cells with a prominent inflammatory infiltrate [17]. It can also show expression of Actin(SM), but the majority also exhibits the reactivity for ALK. Synovial sarcoma is usually composed of two morphologically different types of cells, epithelial cells and spindle cells [18]. Imunohistochemically, the presence of CK and CD99 expression favor the diagnosis of synovial sarsarcoma. Solitary fibrous tumor usually involves the pleura, and is characterized by the random arrangement of the tumor cells and striking hyalinization [19]. The lack of CD34 and CD99 expression in our case can also help to exclude this tumor. Mesothelioma is chracterized by a mixture of epithelial and sarcomatous components with marked hyperchromasia and nuclear pleomorphism [20]. Mesothelioma is usually positive for CK5/6 and Caretinin. Leiomyoma can also be ruled out for lacking Desmin and strong Actin(SM) expression. In addition, all the above tumors were negative for TTF-1, which is a useful marker for differential diagnosis.

## Conclusion

Although PSH is relatively uncommon, it is usually easily diagnosed based on the typical architectural patterns and cell types. However, our case with the pattern of dense spindle cells change may pose a great diagnostic challenge. Using a panel of antibodies including TTF-1 is quite essential for avoiding the misdiagnosis. The biological significance of spindle cells change remains unknown. It is necessary for long-term follow-up.

### Consent

Written informed consent was obtained from the patient for publication of this case report and accompanying images. A copy of the written consent is available for review by the Editor-in Chief of this Journal.

## Competing interests

The authors declare that they have no competing interests.

## Authors’ contributions

**L**XY and WY participated in the histopathological evaluation, performed the literature review, acquired photomicrographs and drafted the manuscript. FCF and LY carried out the immunohistochemical stains evaluation. YJH and DSD conceived and designed the study. WEH gave the final histopathological diagnosis and revised the manuscript. WL edited the manuscript. All the authors read and approved the final manuscript.
